# Tryptophan residue 32 in human Cu-Zn superoxide dismutase modulates prion-like propagation and strain selection

**DOI:** 10.1371/journal.pone.0227655

**Published:** 2020-01-30

**Authors:** Anthony Crown, Luke McAlary, Eric Fagerli, Hilda Brown, Justin J. Yerbury, Ahmad Galaleldeen, Neil R. Cashman, David R. Borchelt, Jacob I. Ayers

**Affiliations:** 1 Center for Translational Research in Neurodegenerative Disease, SantaFe HealthCare Alzheimer’s Disease Research Center, Department of Neuroscience, McKnight Brain Institute, University of Florida, Gainesville, Florida, United States of America; 2 Molecular Horizons and School of Chemistry & Molecular Bioscience, University of Wollongong, New South Wales, Australia; 3 Illawarra Health and Medical Research Institute, School of Chemistry & Molecular Bioscience, University of Wollongong, New South Wales, Australia; 4 Department of Biological Sciences, St. Mary’s University, San Antonio, Texas, United States of America; 5 Djavad Mowafaghian Centre for Brain Health, University of British Columbia, Vancouver, British Columbia, Canada; 6 Institute for Neurodegenerative Disease, University of California, San Francisco, California, United States of America; "INSERM", FRANCE

## Abstract

Mutations in Cu/Zn superoxide dismutase 1 (*SOD1*) associated with familial amyotrophic lateral sclerosis cause the protein to aggregate via a prion-like process in which soluble molecules are recruited to aggregates by conformational templating. These misfolded SOD1 proteins can propagate aggregation-inducing conformations across cellular membranes. Prior studies demonstrated that mutation of a Trp (W) residue at position 32 to Ser (S) suppresses the propagation of misfolded conformations between cells, whereas other studies have shown that mutation of Trp 32 to Phe (F), or Cys 111 to Ser, can act in cis to attenuate aggregation of mutant SOD1. By expressing mutant SOD1 fused with yellow fluorescent protein (YFP), we compared the relative ability of these mutations to modulate the formation of inclusions by ALS-mutant SOD1 (G93A and G85R). Only mutation of Trp 32 to Ser persistently reduced the formation of the amorphous inclusions that form in these cells, consistent with the idea that a Ser at position 32 inhibits templated propagation of aggregation prone conformations. To further test this idea, we produced aggregated fibrils of recombinant SOD1-W32S *in vitro* and injected them into the spinal cords of newborn mice expressing G85R-SOD1: YFP. The injected mice developed an earlier onset paralysis with a frequency similar to mice injected with WT SOD1 fibrils, generating a strain of misfolded SOD1 that produced highly fibrillar inclusion pathology. These findings suggest that the effect of Trp 32 in modulating the propagation of misfolded SOD1 conformations may be dependent upon the “strain” of the conformer that is propagating.

## Introduction

Approximately 10–20% of familial amyotrophic lateral sclerosis (fALS) cases are associated with mutations in the ubiquitously expressed superoxide scavenging cytosolic enzyme Cu-Zn superoxide dismutase (SOD1) [1–3]. SOD1-associated ALS is generally considered to be a “classical” phenotype, which is characterized by loss of upper and lower motor neuron function. The list of mutations in SOD1 that have been associated with ALS is ever growing and currently stands at more than 160 mutations in the ALSoD database [4], which includes mutations that are definitively identified as dominantly inherited as well as private mutations found in isolated cases. The mean age of disease onset for SOD1-fALS patients is 45–47 years [5], whereas the average age of onset in sporadic ALS cases tends to be later (55–60 years of age) [6].

The vast majority of SOD1 mutations listed in the database are missense point mutations [4]. A subset of mutations result in early translation termination, yielding truncated proteins that lack a portion or all of the residues encoded in the 5^th^ and last coding exon. Although these early truncation mutations are clearly catastrophic for enzymatic activity and protein stability, the effect of disease associated point mutations is more variable with some mutations having minimal impact on activity or protein half-life [7–10]. Almost all SOD1-ALS patients are heterozygous for the mutant gene and in patients with mutations that reduce activity or protein stability, some level of reduced enzymatic activity has been observed [11]; however, there is no obvious correlation between residual enzymatic activity and age of disease onset or duration [12]. Only three mutations that would be predicted to produce mRNAs that would be degraded by nonsense mediated decay pathways are reported in the database [4], and the etiological significance of these mutations is uncertain. A pathologic feature of SOD1-linked fALS that is commonly, but not uniformly found, is the accumulation of SOD1 immuno-reactive inclusions in surviving spinal motor neurons [13–28]. Multiple studies have demonstrated that fALS mutant SOD1 expressed in cultured cells is generally more prone to misfold and aggregate than wild-type [5,29–33], and aggregation of mutant SOD1 appears to be a critical factor in disease pathogenesis [32,34,35]. Overall, there is considerable evidence that the misfolding and aggregation of mutant SOD1 is a key event in the toxic processes that produce motor neuron disease (reviewed in [36]).

Misfolded SOD1 has also been described as a pathologic feature of sporadic ALS using antibodies that are more reactive to non-natively folded SOD1 than the natively folded protein [37–39]. However, the frequency of misfolded SOD1 in sporadic ALS as a pathologic feature has been disputed [40–42]. WT SOD1 is highly expressed in motor neurons, approaching supersaturation, which could enhance its probability of misfolding [43]. *In vitro*, purified WT SOD1 can be readily induced to aggregate into amyloid-like fibrillary structures by de-metalation (Cu) and disulfide reduction [44–54]. Within the amino acid sequence of SOD1 there are several small sequence motifs that are inherently amyloidogenic [55]. Moreover, mice that highly over-express WT human SOD1 develop motor neuron disease that is similar to mice expressing mutant SOD1 [56]. Finally, in cell culture models, WT-SOD1 can support the prion-like propagation of misfolded conformations [57,58]. Although there is clearly evidence to support the idea that SOD1 misfolding could be involved in sporadic ALS, consensus regarding misfolded SOD1 in spinal tissues of these patients remains elusive [42].

The misfolding and aggregation of mutant SOD1 exhibits features of prion-like propagation and conformational templating in seeding aggregation [59]. Both WT and fALS mutant SOD1 can be induced to form fibrillary amyloid structures by de-metallation and reduction of the intramolecular disulfide bond within the SOD1 monomer [49,52,60,61]. In *in vitro* assays, using purified protein, some investigators have reported reproducible differences in the aggregation propensity of WT and fALS mutant SOD1 while others have not [47,48,52]. One study of a large number of mutants used purified holo pseudo WT-SOD1, which is a variant in which Cys residues at 6 and 111 are mutated to Ala and Ser to reduce non-specific intermolecular disulfide crosslinking. This variant designated pSOD1, is fully metallated and presumably has an intramolecular disulfide bond between residues 57 and 120. Purified pSOD1 is slow to aggregate *in vitro* (days as compared to hours) and generally forms non-amyloid aggregates [62]. Mutation of pSOD1 with fALS mutations, such as A4V, A4T, G85R, G93A, and I149T, was reported to decrease the lag phase for aggregate formation; however, other fALS mutants such as A4S, G93D, G93V, and G93S showed little or no difference in aggregation rates [62]. Notably, the *in vitro* aggregation of both WT and mutant SOD1 can be significantly accelerated by the addition of minute quantities of pre-formed SOD1 multimeric seeds [63]. Live-imaging studies of cells that express mutant SOD1 fused to the photo-convertible fluorophore Dendra have demonstrated that intracellular aggregates of mutant SOD1 grow through recruitment of both newly-translated and pre-existing pools of soluble precursors [64]. Moreover, *in vivo* prion-like transmission of misfolded SOD1 seeds has been demonstrated by the intraspinal injection of spinal cord homogenates of paralyzed mice into mice that express low levels of the G85R variant of human SOD1 (expressed with or without an in-frame fusion of yellow fluorescent protein)[35,65,66]. Accelerated seeding of mutant SOD1 aggregation *in vivo* by intraspinal injection has also been observed for mice that express the ALS truncation mutant L126Z and an experimental truncation mutant terminating at residue 103 [65]. Importantly, conformational elements in the misfolded SOD1 that populates tissue homogenates can seed SOD1-G85R to deposit as distinct pathological morphologies that propagate through repeated rounds of *in vivo* seeding [65]. Collectively, these data are consistent with the idea that misfolded conformers of mutant SOD1 can template conformational changes to naïve SOD1 molecules by prion-like mechanisms of interaction and aggregation.

Prior studies of ALS mutant SOD1 aggregation have shown that mutation of the unique Trp residue at position 32 of SOD1 to Ser or Phe can act in cis to suppress aggregation [38,67–69]. Modulation of ALS mutant SOD1 aggregation by substitution of Cys 111 to Ser has also been described [70–72]. The mutation of Trp 32 to Ser potently inhibited the ability of misfolded WT SOD1 to propagate between cells in culture [57,68,69]. Small molecules that bind near Trp 32 can block the seeding of mutant SOD1 aggregation in cell culture models [68,69,73]. In the present study, we used a combination of cell culture models and *in vivo* seeding models to examine the role of Trp 32 in modulating mutant SOD1 aggregation. The data suggest that mutation of Trp 32 to Ser affects seeded aggregation of SOD1 in a strain-dependent manner.

## Materials and methods

### Cell transfections with YFP-tagged SOD1

Mutant SOD1-YFP fusion plasmids were constructed by inducing point mutations on template SOD-WT-YFP cDNA with the Quick-Change mutagenesis kit (Cat. No. 200523, Thermo/Fisher, Waltham, MA, purchased 2014). All clones were prepared in a modified version of the pEF-Bos expression vector [74]. The presence of the desired mutation was confirmed by DNA sequence analysis. Cesium chloride purification was used to prepare the verified DNA plasmids for transfection.

SOD-YFP fusion expression plasmids were transfected into Chinese Hamster Ovary cells (ATCC CCL-61). The cells were originally obtained in 2008 from ATCC at which time they were expanded and then frozen in aliquots. The cells have not be authenticated and the maximum passage number for cells used in transfection was 20. Cells were split into 12 well plates and incubated at 37°C, 5% CO_2_ for 24 hours. At 95% confluency, cells were then transiently transfected with 800ng of plasmid DNA and Lipofectamine 2000 (Cat. No. 11668019, Invitrogen/ThermoFisher, purchased in 2014). The transfected cells were then imaged using fluorescence microscopy in an AMG EVOS_fl_ inverted digital microscope. Representative pictures were taken at 24 and 48 hours after transfection at 20x magnification. Each transfection was repeated at least three times.

The number of cells analyzed per experiment is noted in the Figure legends.

### Statistical analysis of cell imaging data

For quantitative analysis of aggregate formation, random images were captured and codified for scoring for the presence of inclusion-like structures by a blinded observer. Cells were scored as having inclusions when the observer could clearly discern the presence of multiple highly fluorescent puncta within the cytosol. Cells expressing the ALS variants G93A and/or G85R were used as positive control for comparisons. Cells that were detached were generally not counted because they were slightly out of focus relative to the flatter cells. No sample size calculation was performed. The number of total cells counted for each construct across the replications averaged between 60 and 213 cells per construct per experiment (see Figure Legends). A two-tailed type-2 t-test was carried out in Excel (Microsoft Office Professional 2016, Microsoft, Redmond, WA) to determine whether the percentage of cells developing inclusions differed between cells expressing individual constructs in a pairwise fashion. No outliers were excluded. Given the number of cells that were counted for each construct, we assumed a normal distribution of the data.

### Immunocytochemistry with C4F6 antibody

For immunocytochemistry, the cells were split onto cover slips (Cat. No. 12546, Fisher Scientific, Waltham MA) that were coated with Poly-D-Lysine (Cat. No. P8920, Sigma Aldrich, St. Louis, MO) the day before transfection. At 24 and 48 hours, the cells were fixed in 4% paraformaldehyde for 10 minutes. Following a brief 1× PBS wash, the fixed cells were permeabilized in ice-cold methanol for 5 minutes. After another wash in PBS-T, a 20% normal goat serum solution in PBS-T was used as means to block the cells for 30 minutes at room temperature. The cells were then incubated for 24 hours at 4°C in the primary antibody, C4F6 (Cat. No. MM-0070-2, Medimabs, Quebec, CA, purchased 2014), which was diluted in a 10% goat serum/PBS-T solution. The cells were washed in PBS-T prior to the addition of goat anti-mouse AlexaFlour-568 (Cat. No. A11004, Invitrogen/ThermoFisher, purchased 2014) secondary antibody and DAPI reagent in dilutions of 1:2000 each in a solution of 10% normal goat serum /PBS-T. The cells were finally washed with PBS and mounted onto glass slides (Cat. No. 1255015, Thermo/Fisher, purchased 2014) using Vectashield mounting medium (Cat. No. H1200, Vector Laboratories, purchased 2014). Pictures were taken at 20× magnification on an Olympus BX60 epifluorescence microscope.

### Recombinant SOD1 production

Expression vectors encoding human SOD1 and the yeast copper chaperone for SOD1 (pACA-yCCS-hSOD1) for bacterial expression were a gift from Professor Mikael Oliveberg (Stockholm University, Sweden). Plasmids encoding W32S constructs were designed in-house and generated by Genscript using site directed mutagenesis (Piscataway, NJ, USA). SOD1 expression and purification was performed as previously described [75,76]. The pACA forward yCCS hSOD1 expression vectors were transformed in BL21(DE3) *E*.*coli*, followed by induction of cultures with isopropyl β D-1thiogalactopyranoside (IPTG) in the presence of CuSO_4_ and ZnSO_4_. Following lysis and SOD1 precipitation using ammonium-sulfate, SOD1 was purified by size exclusion chromatography (Superdex^™^ 200, 10/300 GL, Cat. No. 15-5175-01, GE Healthcare Lifesciences, Pittsburgh, PA, purchased 2015,) and anion exchange chromatography (HiPrep^™^ DEAE FF 16/10, Cat. No. 28-9365-41, GE Healthcare Lifesciences, purchased 2013). SOD1 purity was determined by reducing SDS-PAGE and mass spectrometry with samples then being snap frozen in liquid nitrogen and stored at -20 °C in aliquots for later use. Protein concentration was determined using bicinchoninic acid protein assay.

### SOD1 fibrillization *in vitro*

Purified recombinant SOD1 was demetallated as described previously [77]. Briefly, purified SOD1 was dialyzed in a 10,000 Da Slide-a-lyzer (Cat. No. 69576 or Cat. No. 88404, depending on volume, ThermoFisher, purchased 2013 and 2015) against 100 mM sodium acetate, pH 3.8 overnight, followed by dialyzing against 100 mM sodium acetate, pH 3.8 and 10 mM EDTA overnight. The recombinant SOD1 was then dialyzed in successive solutions of chelexed 100 mM sodium acetate, pH 3.8 overnight and chelexed in 20 mM potassium phosphate, pH 7.0 overnight.

50 μm of apo SOD1 was fibrilized in 20 mM potassium phosphate, pH 7.2 with the addition of 10 mM TCEP. For those samples used for screening fibril formation, 4 μm Thioflavin T was added, whereas no Thioflavin T was added to those samples to be used as inoculum. Two hundred microliters of the protein solutions were incubated in a 96-well plate with the addition of a Teflon ball (1/8-in diameter) at 37°C with constant agitation in a Synergy HT plate reader (BioTek Instruments, Winooski, VT). Fluorescence measurements were recorded every 15 min using a λ_ex_ = 440/30 filter to excite and a λ_em_ = 485/20 filter to detect emission using the Gen5 software (v1.10.8).

The presence of aggregates was confirmed by filter trap assay as previously described [65]. The membrane was then immunoblotted using the hSOD1 antibody at 1:2500 and imaged using the Pxi blot imaging system (Syngene, Frederick, MD).

### Animal inoculations

All studies involving mice were approved by the Institutional Animal Care and Use Committee (IACUC) at the University of Florida (Protocol Number 201508784) in accordance with the NIH guidelines. Mice expressing the G85R-SOD1: YFP transgene were originally obtained from Dr. Arthur Horwich (Yale University, New Haven, CT) [78]. All animals were housed one to five to a cage and maintained on ad libitum food and water with a 14-h light and 10-h dark cycle. Both males and female mice were used for injection. Spinal injection in neonatal mouse pups were performed as previously described [35]. Briefly, P0 neonatal G85R-SOD1: YFP mice on the FVB/NJ background were cryoanesthetized and injected with 1 μl of inoculum, slowly in the vertebral column. After the procedure, mice were monitored to ensure full mobility and no signs of impairment. All animals fully recovered from the injections and no medications were required. Entire litters of newborn G85R-SOD1: YFP mice were chosen at random for injection with a single inoculum. Otherwise, no randomization was performed to allocate subjects in the study.

### Tissue collection and fluorescence microscopy

Mice that reached an age endpoint, or exhibited bilateral hindlimb paralysis, were euthanized by anesthetization with isoflurane, which is a fast acting inhaled anesthetic that is approved for use in mice. Once animals were non-responsive to foot-pinch, the chest cavity was opened and the animal was exsanguinated by transcardial perfusion with 20 ml of PBS, and the brain and spinal cord were immediately removed and placed in dry ice, for biochemical studies, or in 4% paraformaldehyde for 24–48 h at 4 °C. Fixed tissue was then impregnated with 30% sucrose in PBS, mounted in OCT media (Cat. No. 4583, Sakura Finetek, The Netherlands), sectioned to 30 μm using a cryostat, and placed in a dish containing anti-freeze solution (100 mM sodium acetate, 250 mM polyvinylpyrrolidone, and 40% ethylene glycol) at pH 6.5. Sections were then mounted onto slides, air-dried overnight, and coverslipped in mounting media containing DAPI (Cat. No. H1200, Vector Laboratories, Burlingame, CA, purchased 2014). For some experiments, we used an Olympus DSU-IX81 Spinning Disc Confocal microscope, controlled by Slidebook software (v4.2, Intelligent Imaging Innovations, Inc, Denver, CO). We also used a laser-scanning confocal microscope Nikon A1RMP image system with a Nikon A1R scanner, Nikon A1-DUG 4 channel filter-based detector unit and Nikon LUNV Laser Launch (6 lines) with a 40× objective, or a 60× water immersion objective. The excitation/emission wavelengths during acquisition were 488 nm/492–557 nm for GFP. Images were processed using Nikon NIS-Elements software v4.5 (Nikon Instruments, Inc., Melville, NY).

## Results

### *In vivo* induction of motor neuron disease by transmission of recombinant W32S-SOD1 fibrils

**Fig 1:**
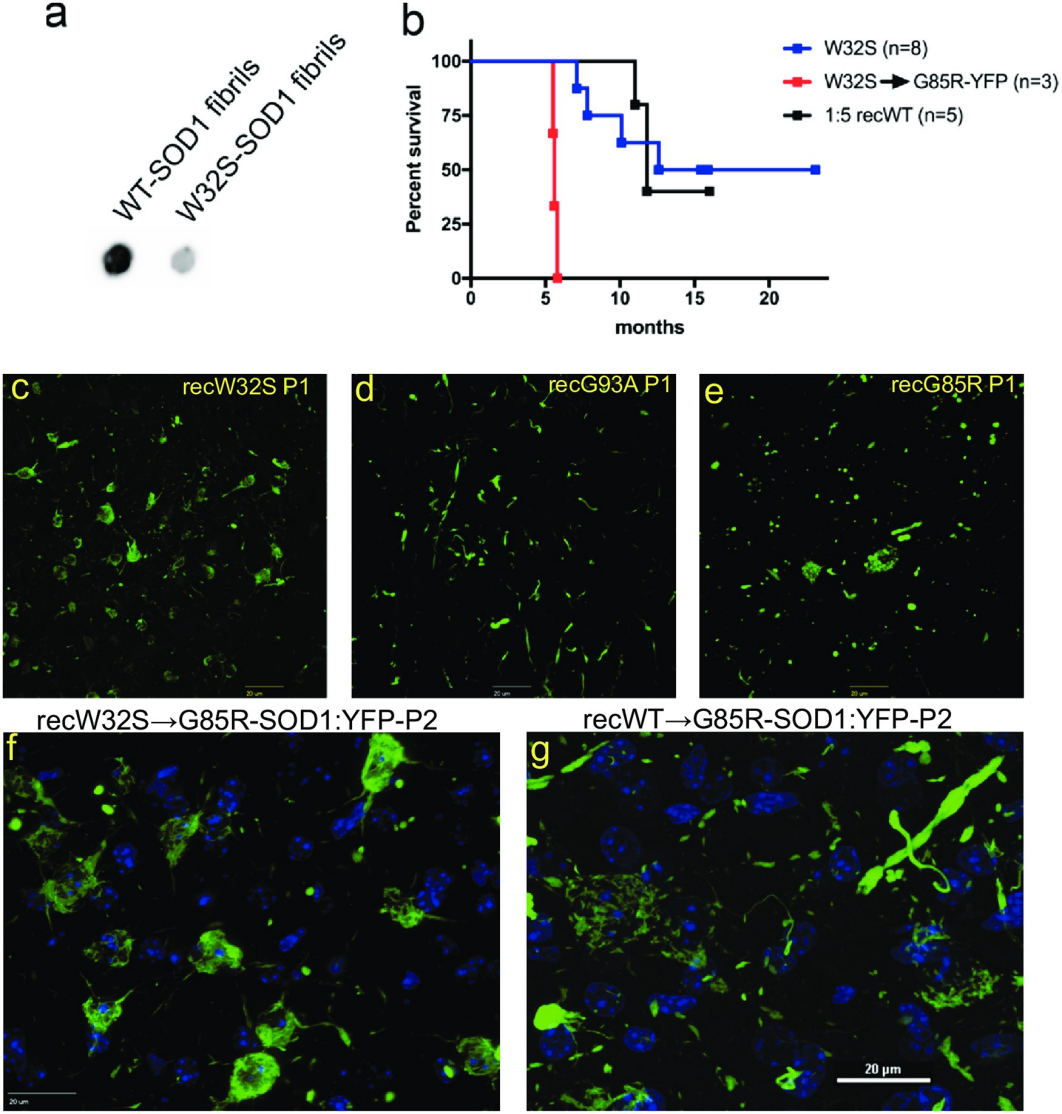
Recombinant W32S-SOD1 fibrils induced disease in G85R-SOD1: YFP transgenic mice. A) *In vitro* fibrilized recombinant SOD1 was quantified by filter trap assay (n = 3). B) Fibrils of W32S-SOD1 or WT-SOD1 (diluted 5-fold) were injected into the spinal cords of G85R-SOD1: YFP mice at postnatal day P0. Upon disease induction in a G85R-SOD1: YFP mouse injected with W32S-SOD1 fibrils, the spinal cord from this mouse was homogenized and injected into naïve G85R-SOD1: YFP mice (W32S → G85R-YFP). C-E) G85R-SOD1: YFP inclusion pathology was visualized at the endstage of disease in mice injected in the spinal cord at postnatal day P0 with recombinant fibrils as indicated in the figure. F-G) Inclusion pathology in G85R-SOD1: YFP mice inoculated with 2^nd^ passage spinal homogenates from mice initially injected with WT or W32S SOD1 fibrils. Scale bars in panels C-G are 20 μm. The images in panels C-E were captured at 40 x on a Nikon confocal microscope. The images in F and G were captured at 60 x on either a Nikon confocal or an Olympus spinning disk confocal, respectively.

In previous studies, we have demonstrated the ability to induce motor neuron disease (MND) in mice that express G85R-SOD1: YFP at low levels by injecting recombinant WT SOD1 that had been fibrilized *in vitro* [65]. To determine whether the W32S substitution could inhibit the *in vivo* seeding of G85R-SOD1: YFP aggregation, we prepared recombinant WT-SOD1 and W32S-SOD1 fibrils as described in Methods (S1 Fig). To standardize for the amount of fibrils produced in the assay, we assessed the levels of large aggregates by filter trap assay and observed a ~5 fold greater level WT-SOD1 fibrils than W32S-SOD1 fibrils in our preparations (Fig 1a). We therefore diluted the WT-SOD1 fibrils 5-fold and injected this preparation, along with undiluted W32S-SOD1 fibrils, into the spinal cords of G85R-SOD1: YFP mice at postnatal day P0 to test for MND induction. We injected the diluted WT-SOD1 fibrils into 5 G85R-SOD1: YFP mice, with 3 developing MND with an average time to end-stage of 11.53 ± 0.5 months (Table 1, Fig 1b). Of the 8 G85R-SOD1: YFP mice injected with W32S-SOD1 fibrils, 4 developed MND with an average time to paralysis of 9.4 ± 1.2 months (Fig 1b). The other 4 mice were aged to at least 15 months p.i. and developed no signs of paralysis (Table 1). We performed a second passage by injecting a naïve litter of P0 G85R-SOD1: YFP mice with spinal cord homogenate prepared from paralyzed mice that had received the W32S-SOD1 injections. From these injections, all 3 G85R-SOD1: YFP injected mice developed MND with a time to disease end-stage of 5.6 ± 0.1 months. For comparison, we also generated fibrils from recombinant SOD1 encoding the G93A and G85R mutations associated with ALS. These preparations also induced accelerated MND with the G93A preparation showing the highest efficacy (Table 1). Overall, the ability of the W32S fibrils to induce MND and seed G85R-SOD:YFP inclusion pathology appeared to be relatively similar to WT or ALS mutant SOD1 fibrils.

**Table 1:** Transmissibility of recombinant SOD1 fibrils by intraspinal injection into newborn G85R-SOD1: YFP mice.

rec SOD1 protein	Fibrils visible by EM	Induced Paralysis in P0 injected G85R:YFP mice (age range)
WT (1:5)	yes	3 / 5 (11.0–11.8 mo) (2 disease-free @ 16 mo)
W32S	yes	4 / 8 (7.1–12.6 mo (4 disease-free @ 16mo)
G85R	not done	6 / 7 (8.4–15.9) (1 disease-free @ 16 mo)
G93A	yes	10 / 10 (8.0–15.0 mo)

The spinal cords of paralyzed mice injected with W32S-SOD1 fibrils displayed an abundance of skein-like inclusions primarily localized within neuronal cell bodies (Fig 1c). By contrast, the inclusion pathology of paralyzed G85R-SOD1: YFP mice injected with G93A or G85R SOD1 fibrils was localized primarily in the neuropil. In the mice injected with the G93A fibrils, the inclusions appeared to be long fibrils (Fig 1d) whereas in mice injected with the G85R fibrils the inclusions appeared more punctate (Fig 1e). The skein-like inclusion pathology induced by recombinant W32S SOD1 fibrils was also observed in paralyzed mice inoculated with the second-passage spinal homogenates (Fig 1f). The spinal cords of paralyzed mice injected with second passage spinal homogenates from mice initially inoculated with WT-SOD1 fibrils also produced neuronal skein-like inclusions, but there were also abundant fibrillar inclusions distributed in the neuropil (Fig 1g; also see [41]). Altogether, these findings suggest that a fibrillar aggregate of SOD1 with the W32S substitution is capable of supporting conformational templating to induce the misfolding of G85R-SOD1: YFP *in vivo*, producing a misfolded conformer that adopts distinct pathological morphologies.

### Effects of the W32S-SOD1 mutation on SOD1 aggregation in an *in vitro* cell model

**Fig 2:**
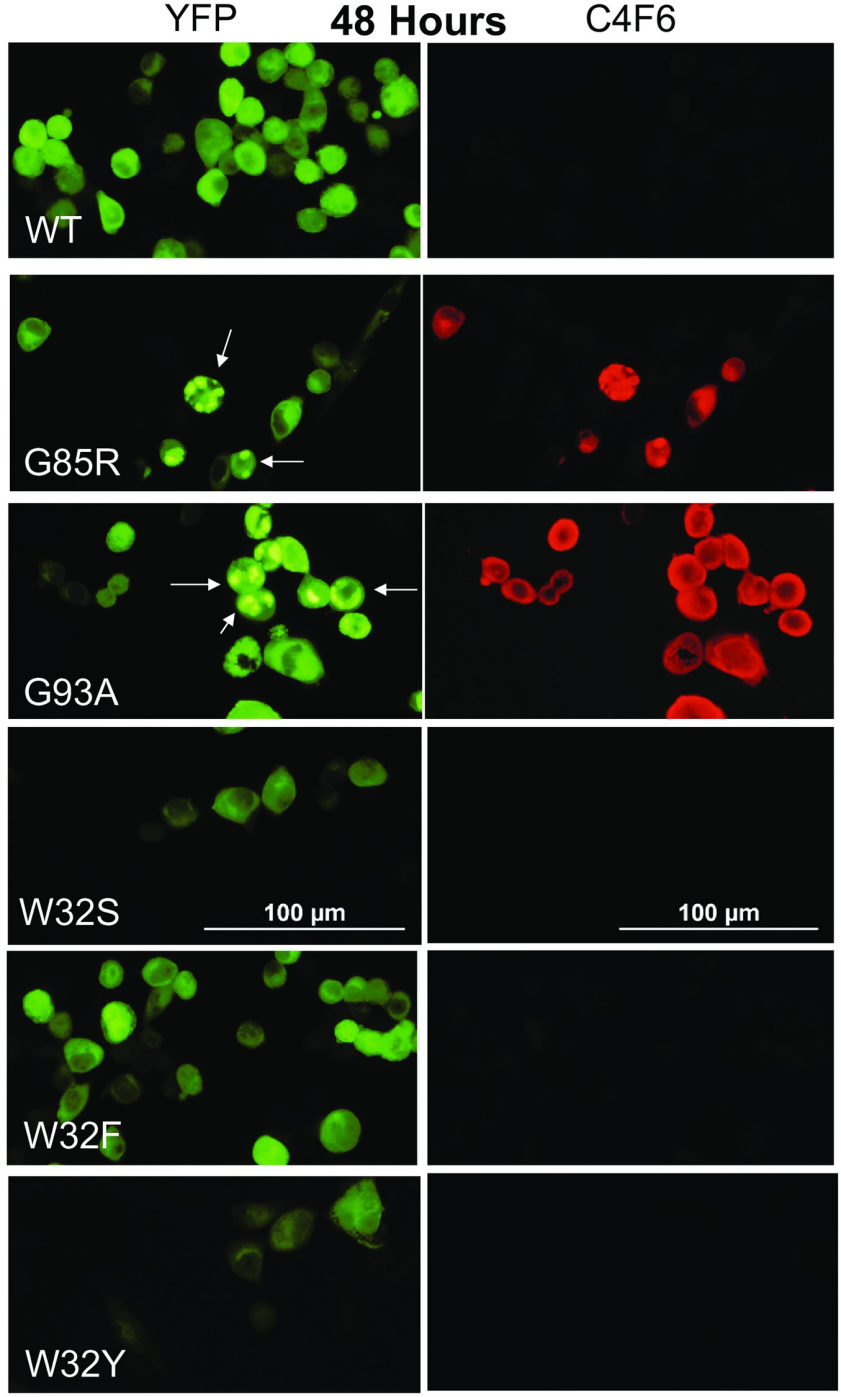
Aggregation assay of SOD1 with single mutant variations at Trp 32. CHO cells were transiently transfected with WT-SOD1: YFP and compared to SOD1: YFP with mutations of Trp 32 to Ser, Phe, and Tyr. Cells were fixed and immunostained with C4F6 antibody at 48 hours post-transfection. As compared to positive control cells expressing G85R and G93A-SOD1: YFP, we observed no aggregation of the Trp 32 mutants and no reactivity to C4F6. The images shown are representative of images captured from 3 independent transfection experiments. The number of cells analyzed per experiment ranged from 45 to 171. Examples of cells transfected with G85R or G93A SOD1: YFP that scored has having inclusions are marked by arrows.

To determine the ability for amino substitutions at tryptophan 32 (W32) to suppress aggregation of ALS mutant SOD1, we employed a cell model system that we have used in prior studies [71,79–81]. We generated SOD1: YFP fusion constructs as described in Methods and transfected these into Chinese Hamster Ovary (CHO) cells. CHO cells were chosen due to their flat morphology, which allowed for clear depictions of the localization of fluorescent protein and intracellular inclusions. In these over-expression paradigms, any cell specific factors that could modulate aggregation, such as chaperones, are overwhelmed and the model provides a reasonable estimate of the inherent aggregation tendencies the protein [82]. Initially, we generated 3 variants of SOD1 with substitutions of W32 in order to compare each single mutation (W32S, W32F, W32Y) against wild type SOD1: YFP fusion. The W32F and W32Y mutations were generated to preserve the aromatic side-chain for comparison. None of these substitutions induced aggregation and inclusion formation when examined 24 or 48 hours post-transfection (S2 Fig). In order to examine the effect of W32 substitutions on SOD1 folding, we performed immunocytochemistry using the C4F6 antibody, which when used in immunocytochemistry in fixed cells appears to recognize an altered conformation that is specific for mutant SOD1 [41]. In prior studies of cells expressing A4V-SOD1: YFP, we noted that C4F6 reactivity to inclusion structures that formed was variable; however, strong reactivity to diffusely distributed mutant protein was observed [82]. We observe a similar pattern here in that the inclusions formed by G93A-SOD1: YFP were weakly reactive (Fig 2, arrows) as compared to the diffusely distributed protein producing diffuse YFP fluorescence. C4F6 was also strongly reactive to cells expressing G85R-SOD1: YFP, but not to cells expressing WT-SOD1: YFP or cells expressing W32S-, W32F-, or W32Y-SOD1: YFP (Fig 2; 24 h shown in S3 Fig). These data indicate that W32 substitutions do not induce conformational changes associated with FALS mutations.

**Fig 3:**
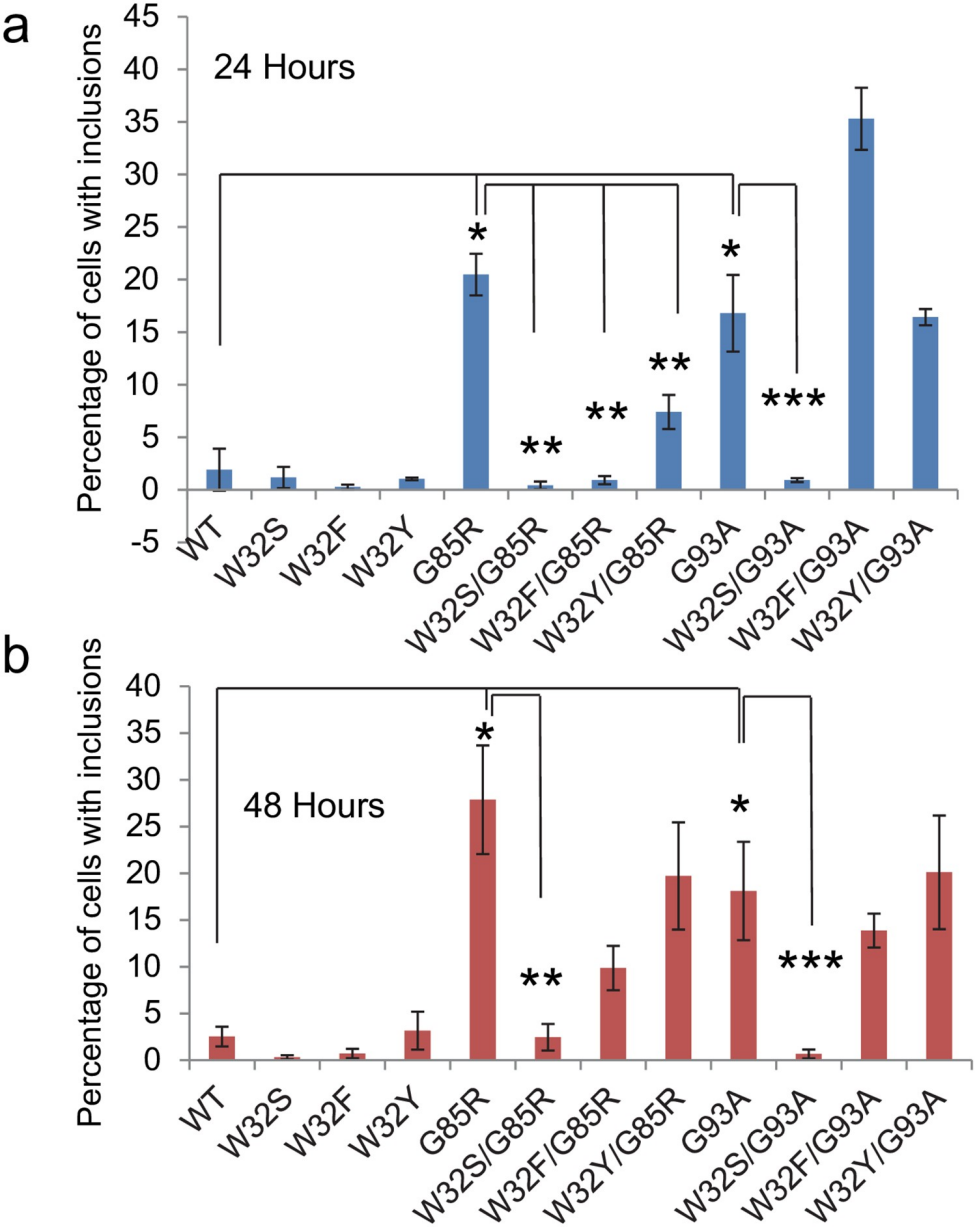
Quantification of SOD1: YFP aggregation at 24 and 48 hours post-transfection. CHO cells were transiently transfected with expression vectors for the various SOD1: YFP constructs and images of the cells were captured at 24 and 48 hours (see S3 and S4 Figs). The effects of substitutions at Trp 32 on SOD aggregation were compared to constructs expressing either G85R or G93A-SOD1: YFP. The data for transfection with WT-, G85R-, and G93A-SOD1: YFP are averages from 6 independent experiments. All other data are from three independent transfections. For all experiments, random images were captured and examined by an observer blind to genotype. The number of total cells counted for each construct across the replications averaged between 60 and 213 cells per construct per experiment. A two-tailed type-2 t-test was used to determine whether the percentage of cells developing inclusions differed between cells expressing individual constructs in a pairwise fashion. *p<0.05 for comparison of cells expressing WT to cells expressing G85R- or G93A-SOD1: YFP. **p<0.05 for comparison of cells expressing G85R-SOD1: YFP to cells expressing W32S/G85R-, W32F/G85R-, or W32Y/G85R-SOD1: YFP. ***p<0.05 for comparison of cells expressing G93A-SOD1: YFP to cells expressing W32S/G93A-, W32F/G93A-, or W32Y/G93A-SOD1: YFP.

We then generated double mutants in which FALS mutations (G85R or G93A-SOD1: YFP) were each combined with the W32 substitution. Images of transfected cells were captured at 24 hours using fluorescence microscopy (S4 and S5 Figs). The presence of inclusion structures amongst all fluorescing cells was then quantified to determine the percentage of cells that harbor aggregates. At 24 hours post-transfection, the combination of W32S, W32F, or W32Y with G85R produced reductions (> 2.5-fold reduction) in the percentage of cells with inclusions (Fig 3A). For the G93A mutation, only the W32S substitution suppressed inclusion formation by ~15-fold (Fig 3A). At 48 hours post-transfection, only the W32S substitution in either G85R or G93A-SOD1: YFP significantly reduced the percentage of cells forming inclusions (Fig 3B). Collectively, the data indicate that the W32S substitution robustly and persistently suppresses inclusion formation by G85R- or G93A-SOD1: YFP.

**Fig 4:**
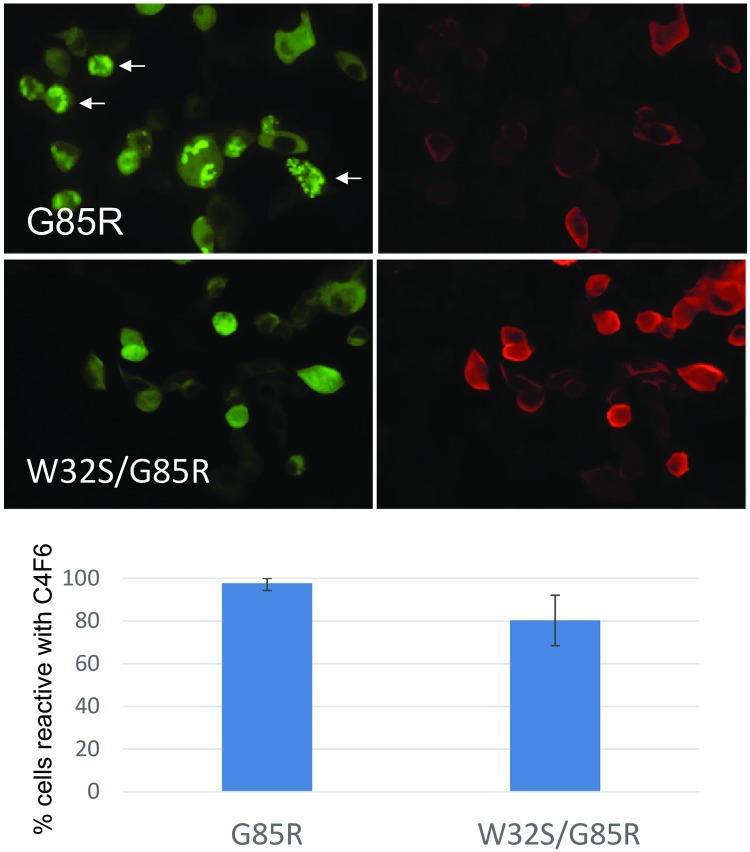
The W32S/G85R-SOD1: YFP retains reactivity with the conformational antibody C4F6. CHO cells were transfected and after 48 hours they were fixed and then immunostained as described in Methods. The single mutant G85R-SOD1: YFP was transfected alongside as a positive control. Three separate transfection experiments were performed and after immunostaining the percentage of cells expressing G85R-SOD1: YFP and W32S/G85R:SOD1: YFP that were reactive to C4F6 was determined by an observer blind to genotype. The total number of cells counted for each construct was ~300. No significant difference in C4F6 reactivity between the cells expressing these YFP fusion constructs was detected. Examples of cells transfected with G85R-SOD1: YFP that scored has having inclusions are marked by arrows.

To determine whether the W32S mutation mitigates the misfolding caused by the G85R mutation, we examined the relative ability of G85R and W32S-G85R-SOD1: YFP to bind the C4F6 antibody. We focused on the G85R variant because the C4F6 antibody was raised against G93A SOD1 [83] and the epitope for the antibody spans residues 90 to 96 and includes the mutated residue [41,84]. Reactivity of the G85R mutant with C4F6 is more reflective of conformational changes near the Gly residue at 93 to expose the epitope for antibody binding. In this experiment, the inclusions formed by G85R-SOD1: YFP were weakly reactive (Fig 4, arrows) as compared to the diffusely distributed protein producing diffuse YFP fluorescence. The double mutant W32S-G85R:SOD1: YFP was similarly highly reactive with the C4F6 antibody (Fig 4), and quantification of images from these cells demonstrated no significant difference in the percentage of cells that were C4F6 reactive relative to cells expressing G85R-SOD1: YFP (Fig 4). These data indicated that although the W32S substitution decreased the formation of inclusions by G85R-SOD1: YFP, the misfolding caused by the G85R mutation that exposes Gly 93 to C4F6 binding was not completely mitigated.

### C111S-SOD1 mutation is unable to suppress SOD1 aggregation in an in vitro cell model

Amino acid substitutions at Cys 111 have also been shown to modulate the aggregation of SOD1, particularly mutation of Cys 111 to Ser [70–72]. To compare the effect of C111S to W32S, we performed aggregation assay studies with constructs, substituting Cys 111 for Ser (C111S) in WT, G93A, and G85R, SOD-YFP fusion plasmid DNA. These constructs were then transiently transfected into CHO cells in triplicate, with representative pictures taken at 24 and 48 hours (S6 Fig). After scoring for presence of aggregates and tabulating a total percentage of cells with aggregates amongst total fluorescing cells, the data were graphed and analyzed by t-test (S7 Fig). These data showed that the C111S substitution did not suppress inclusion formation by the G85R or G93A variants at either 24 or 48 hours post-transfection.

## Discussion

In the present study, we have investigated the role of a Trp residue at position 32 in SOD1 in modulating its ability to propagate and template aggregation. Previous studies have reported that amino acid substitutions at Trp 32 can act in *cis* to suppress the aggregation of ALS mutant SOD1[57,67]. In cell models of SOD1 misfolding and propagation, mutations of Trp 32 to Ser produced dramatic reductions in the ability of SOD1 to be seeded by exogenous mutant SOD1 aggregates [57]. Somewhat surprisingly, we observed that recombinant SOD1 encoding the W32S variation induced MND and induced inclusion pathology when injected into the spinal cords of newborn G85R-SOD1: YFP mice. We further observed that these inclusions were morphologically distinct from the pathology induced by G93A or G85R SOD1 fibrils. While the inclusions in G85R-SOD1: YFP mice injected with the W32S fibrils resembled the pathology of animals injected with WT fibrils, there were also distinctive features. In this study, we also used a paradigm in which the aggregation of ALS-mutant SOD1 was monitored by visualizing the formation of inclusions in cells expressing SOD1 variants fused to YFP. This method is not only technically very simple, but the level of fluorescence of the YFP tag allows easy assessment of whether similar levels of expression have been achieved. We show that experimental mutation of Trp 32 to Phe, Tyr, or Ser does not induce SOD1: YFP to form inclusions or increase the reactivity of SOD1 with C4F6 antibody. When we combined each of these mutations with either the G85R or G93A ALS mutations in *cis*, only the mutation of Trp 32 to Ser produced a robust and persistent reduction in the percentage of cells that developed inclusions. Mutation of Trp 32 to Phe in the context of either G85R or G93A ALS mutations reduced aggregation at 24 hours post-transfection, but not at 48 hours. Mutation of Trp 32 to Tyr modestly suppressed aggregation of G85R-SOD1: YFP but had no effect on G93A-SOD1: YFP. Interestingly, although the Trp 32 Ser mutation suppressed inclusion formation by G85R-SOD1: YFP, this protein was highly reactive to C4F6 antibody. This finding indicates that W32S-G85R-SOD1: YFP is at least partially misfolded such that Gly 93 is exposed to enable C4F6 binding [41]. Our results support the hypothesis that substitution of Trp 32 to Ser somehow uniquely disrupts the assembly of misfolded mutant SOD1 into inclusion-like structures.

Previous work identified several amyloidogenic segments in the SOD1 amino acid sequence, one of which encompasses W32 (^30^KVWGSIKGL^38^) [55]. It is possible that this segment is responsible for the nucleation of aggregates in cultured cells and that the W32S substitution disrupts this fibrillation propensity, an idea supported by the lack of visible aggregates in our cell culture model. Although in cell culture the W32S mutation was capable of blocking inclusion formation in *cis*, we discovered recombinant W32S SOD1 purified from *E coli* could be made to aggregate *in vitro* to produce Thioflavin-positive fibrils. These fibrils potentially nucleated through a different sequence segment [55], resulting in fibrils with a different morphology that could explain the observed G85R-SOD1: YFP aggregate morphology differences between mice inoculated with either WT-SOD1 or W32S-SOD1 fibrils.

The differences in *in vitro* and in-cell nucleation and aggregation could be explained by the different environments of the assays. One aspect of the *in vitro* SOD1 fibrillation protocol is that a reducing agent, such as TCEP, must be present to prevent the formation of amorphous aggregates through random disulfide oxidation. A side effect of TCEP is that it will reduce the normal intramolecular disulfide bond that stabilizes native structure, causing the protein to monomerize, unfold, and aggregate [85,86]. Additionally, unlike the crowded cellular environment W32S-SOD1 faces in the cell culture experiments, in which proteases, chaperones and a multitude of other protein modifying enzymes can affect its folding, the *in vitro* assay is a cell-free environment and lacks these selective pressures. Our ability to generate fibrillar aggregates of W32S-SOD1 set the stage for allowing us to assess whether the Ser substitution might affect the *in vivo* seeding of mutant SOD1 aggregation.

In prior studies, we have shown that transgenic mice expressing G85R-SOD1: YFP at low levels can be induced to develop MND by the injection of spinal tissues harvested from paralyzed mice expressing the G93A, G37R, or L126Z mutations of SOD1 that cause ALS [65]. Accelerated MND could also be induced in these mice by injection of recombinant WT SOD1 that had been incubated to produce Thioflavin-positive fibrillar aggregates [65]. To assess whether the W32S substitution might also attenuate the ability of recombinant SOD1 fibrils to penetrate the CNS and induce the misfolding of mutant SOD1, we fibrilized recombinant W32S-SOD1 *in vitro*. The injection of W32S-SOD1 fibrils into the spinal cords of new born G85R-SOD1: YFP mice produced MND with abundant inclusion pathology at a relatively high frequency. The morphology of pathologic inclusions observed in G85R-SOD1: YFP seeded with W32S fibrils appeared to be distinct from what we have previously seen by injection of WT SOD1 fibrils [65]. Notably, in previous work we observed that the morphology of pathologic inclusions produced by WT SOD1 fibrils was distinct from what was induced by injection of spinal homogenates from G93A mice [65]. Here, we also observed distinct pathologies in mice injected with recombinant fibrils prepared with the G93A or G85R SOD1 mutations. Interestingly, the pathology of G85R-SOD1: YFP mice injected with recombinant G93A fibrils was distinct from what we had previously observed when spinal homogenates of paralyzed G93A mice were used to seed MND [65]. In the latter case, the inclusion pathology showed a punctate morphology within the neuropil [65]; whereas, the recombinant fibrils of G93A protein induced inclusions with an elongated fibril-like appearance. Collectively, these data are consistent with the idea that the G85R variant of SOD1 is susceptible to conformational templating in which distinct misfolded conformers are propagated to produce molecules that assemble in pathologically distinct inclusions. When the seed used to induce MND in this model encodes the W32S mutation, we observe little impact on the ability of the seed to template the misfolding of G85R-SOD1: YFP.

The concept that misfolded SOD1 can adopt multiple conformers is akin to the manifestation of distinct strains of prions. Mice that express different variants of SOD1 accumulate misfolded forms of the protein that exhibit unique signatures of antibody reactivity [87] that are retained when used to induce MND by prion-like transmission to mice expressing G85R-SOD1 [66]. In mice that express G85R-SOD1 fused by YFP, the evidence for strains of misfolded mutant SOD1 manifests by distinctive morphologies of the inclusion pathology that forms initially, and the ability of this distinctive pathology to persist after serial passage to naïve G85R-SOD1: YFP mice [65]. The existence of distinct strains of misfolded SOD1 is a complicating factor in interpreting the outcomes of our study. The observed ability of recombinant W32S-SOD1 to seed the misfolding of G85R-SOD1: YFP *in vivo* may be unique to the strain of misfolded SOD1 conformer that was generated *in vitro*.

The observation that the W32S mutation does not reduce the binding of G85R-SOD1: YFP by the C4F6 antibody suggests that the conformation of the protein around residues 90–96 remains open and accessible. The W32S substitution may suppress the aggregation of G85R or G93A-SOD1: YFP by inhibiting macromolecular interactions in the assembly of larger aggregates. The Trp 32 residue has been suggested to play a role in protein-protein interactions between wild type and mutant G85R SOD1 polypeptides [38]. Other studies have suggested that oxidative modification of Trp 32 could play a role in the misfolding of mutant SOD1 [67,88]. Notably, SOD1 dimers cross-linked at W32 by oxidative modification have also been isolated *in vitro* [88]. Our data are consistent with the view that aromatic oxidation could be an important step in the formation of aggregates, since replacing Trp 32 with aromatic amino acids that oxidize more slowly, Phe and Try, decreased the percentage of cells with inclusions at 24 hours whereas as replacement of Trp 32 with Ser, a non-aromatic amino acid, essentially blocked inclusion formation.

The role of the Cys residue at position 111 of human SOD1 has similarly been the subject of previous study in the aggregation of mutant SOD1. It has been proposed that peroxidation at Cys 111 promotes formation of insoluble aggregates by inducing conformational changes and dissociating the protein’s normal homodimer into oligomeric units [89]. It has also been proposed that oxidation of Cys 111 could produce aberrant intermolecular disulfide bonds that promote aggregation [47,72]. Mice expressing the H46R ALS mutation with mutation of Cys 111 to Ser showed delayed disease onset relative to mice expressing H46R SOD1 [90]. In prior studies, Karch and Borchelt observed that mutating Cys 111 to Ser in human G85R SOD1 dramatically reduced its ability to form detergent insoluble aggregates within 24 hours [70]. However, our study here demonstrates that the suppressive effect of the Cys 111 mutation to Ser was not apparent in our visual assay of G85R-SOD1: YFP aggregation. It is notable that the addition of the YFP tag to the C-terminus of SOD1 is probably not entirely benign even though WT SOD1 fused to YFP appears to be soluble [82] and SOD1 fused to GFP appears to be fully active and correctly dimerized [91]. It is possible that the combined effects of the YFP tag with the G85R mutation impose too great of an impact on SOD1 folding and aggregation to be mitigated by the mutation Cys 111 to Ser. Within the constraints of our assay system, our data suggest that the modification of Trp 32 to Ser is most efficacious in suppressing the assembly of mutant SOD1 into inclusion-like aggregates.

While we lack a precise understanding of how SOD1 aggregation causes the symptoms of fALS, our studies are consistent with the idea that oxidative modification of Trp 32 could play a role in promoting the misfolding and aggregation of mutant SOD1 [67]. Our studies also lend further credence to the hypothesis that SOD1 aggregation may involve a templating mechanism, in which aberrant folded mutant SOD1 protein interacts with either naïve or more natively folded protein to promote aggregation [38]. Further studies of the mechanism by which Trp 32 in SOD1 mediates the assembly of mutant SOD1 into larger aggregates may reveal potential avenues for therapy. Indeed, a recent study demonstrated that 5-fluoruridine, a compound that binds to SOD1 in a pocket that includes Trp 32, can attenuate the induced aggregation of SOD1:GFP in cultured cell models [73]. It may be of interest to generate transgenic mice that express mutant SOD1 with Trp 32 mutations to further explore the role of this residue in the macromolecular interactions that produce mutant SOD1 aggregates.

## Supporting information

S1 Fig Thioflavin T curves of recombinant SOD1 fibril production. Apo WT SOD1 and apo W32S SOD1 were fibrilized in the presence of a reducing agent at 37°C with constant agitation. Thioflavin T fluorescence was measured every 15 minutes for 16 hours. Both proteins revealed an increase in fluorescence over time indicating the formation of SOD1 fibrils.
(TIF)

S2 Fig Aggregation assay for single mutant W32 SOD1: YFP. To determine if SOD1 can withstand substitutions at tryptophan 32 without a change in aggregation propensity, CHO cells were transiently transfected with plasmids for the overexpression of WT, W32F, W32Y and W32S SOD1-YFP. The cells were then imaged using fluorescence microscopy 24 and 48 hours after transfection. The images shown are representative pictures taken from 3 independent experiments. No significant change in the attenuation of aggregates was observed.
(TIF)

S3 Fig Comparisons of SOD1: YFP expression versus C4F6 structural antibody activity on W32 substitutions on SOD1. The cells were fixed and stained 24 hours after transfection as described in the methods section. Wild type SOD1: YFP and mutant G85R SOD1: YFP served as a negative and positive controls respectively. The images shown are representative of 3 independent experiments. Mutations on the codon for SOD1 tryptophan position 32 show virtually no C4F6 selectivity, suggesting SOD1 can withstand mutations at tryptophan 32 without misfolding.
(TIF)

S4 Fig Representative images for G85R SOD1: YFP double mutant aggregation. In order to ascertain if mutations at tryptophan 32 are capable of modulating inclusion formation in misfolded SOD1, plasmids encoding the various G85R SOD1: YFP single and double mutations were transiently transfected into CHO cells. Images were taken using fluorescence microscopy at 24 and 48 hours after transfection and subsequently quantified (see Fig 2). The images depicted are representative images from 3 independent experiments.
(TIF)

S5 Fig Representative images for G93A SOD1-YFP double mutant aggregation assay. Plasmids for expression of either single or double mutant G93A SOD1: YFP were transiently transfected into CHO cells and then imaged at 24 and 48 hours using fluorescence microscopy. Images were then quantified for changes in tendency for inclusion formation (See Fig 2). The images depicted are representative of 3 independent experiments.
(TIF)

S6 Fig C111S aggregation assay representative images. CHO cells were transfected with the various C111S single and double mutant SOD1: YFP cDNA constructs and then imaged 24 and 48 hours after transfection with fluorescence microscopy. Images were then quantified for the presence or absence of aggregates. The images shown are representative images from 3 independent experiments. No observable change in inclusion formation from G85R and G93A single mutant controls was observed.
(TIF)

S7 Fig Analysis of C111S mutation on the aggregation of G85R and G93A-SOD1: YFP. The ability of C111S to suppress aggregation of ALS mutant SOD1 was examined by transient transfection of CHO cells. The data for transfection with WT-, G85R-, and G93A-SOD1: YFP are averages from 6 independent experiments. All other data are from three independent transfections. For all experiments, random images were captured and examined by an observer blind to genotype (S5 Fig for examples of images). The number of total cells counted for each construct across the replications averaged between 79 and 175 cells per construct per experiment. A two-tailed type-2 t-test was used to determine whether the percentage of cells developing inclusions differed between cells expressing individual constructs in a pairwise fashion. The introduction of the C111S substitution to G85R or G93A-SOD1: YFP did not significantly reduce the number of cells that produced inclusions.
(TIF)
