# Supplementary figures and images for: Tryptophan residue 32 in human Cu-Zn superoxide dismutase modulates prion-like propagation and strain selection
Source: PLoS One. 2020 Jan 30;15(1):e0227655. doi: 10.1371/journal.pone.0227655 (PMC6991973; doi:10.1371/journal.pone.0227655)

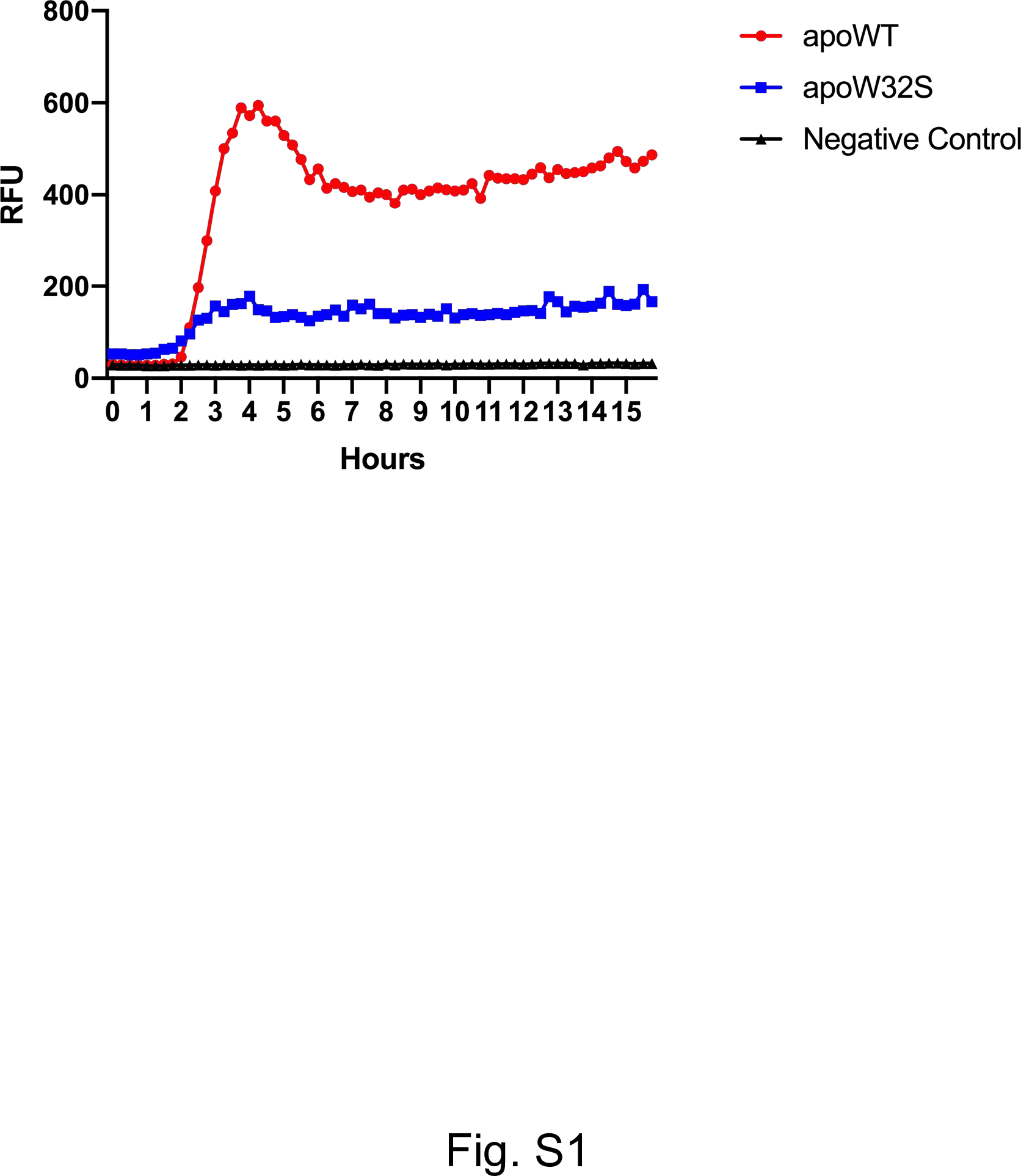

Supplement: S1 Fig — Apo WT SOD1 and apo W32S SOD1 were fibrilized in the presence of a reducing agent at 37°C with constant agitation. Thioflavin T fluorescence was measured every 15 minutes for 16 hours. Both proteins revealed an increase in fluorescence over time indicating the formation of SOD1 fibrils. (TIF) [file pone.0227655.s001.tif]

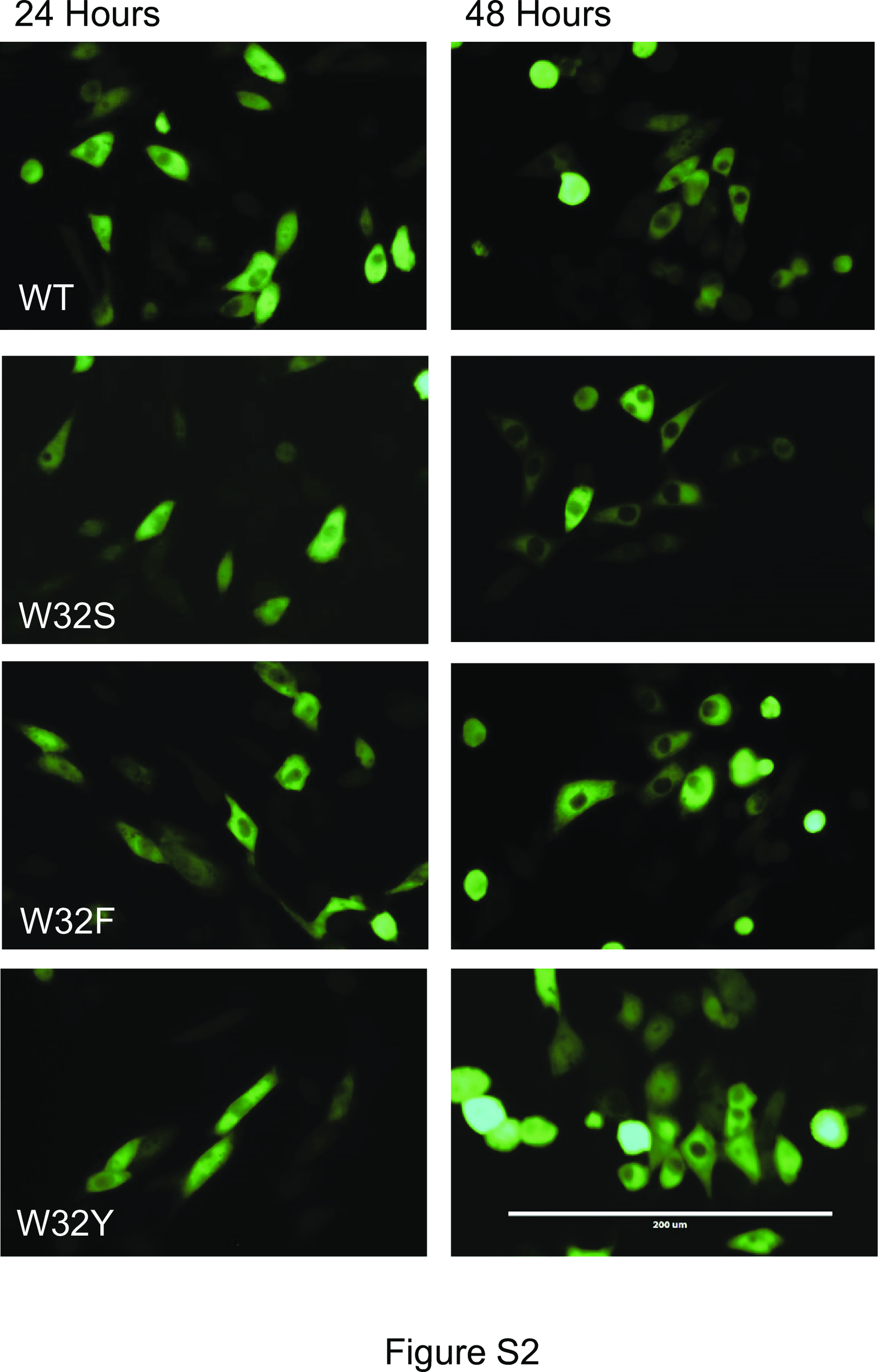

Supplement: S2 Fig — To determine if SOD1 can withstand substitutions at tryptophan 32 without a change in aggregation propensity, CHO cells were transiently transfected with plasmids for the overexpression of WT, W32F, W32Y and W32S SOD1-YFP. The cells were then imaged using fluorescence microscopy 24 and 48 hours after transfection. The images shown are representative pictures taken from 3 independent experiments. No significant change in the attenuation of aggregates was observed. (TIF) [file pone.0227655.s002.tif]

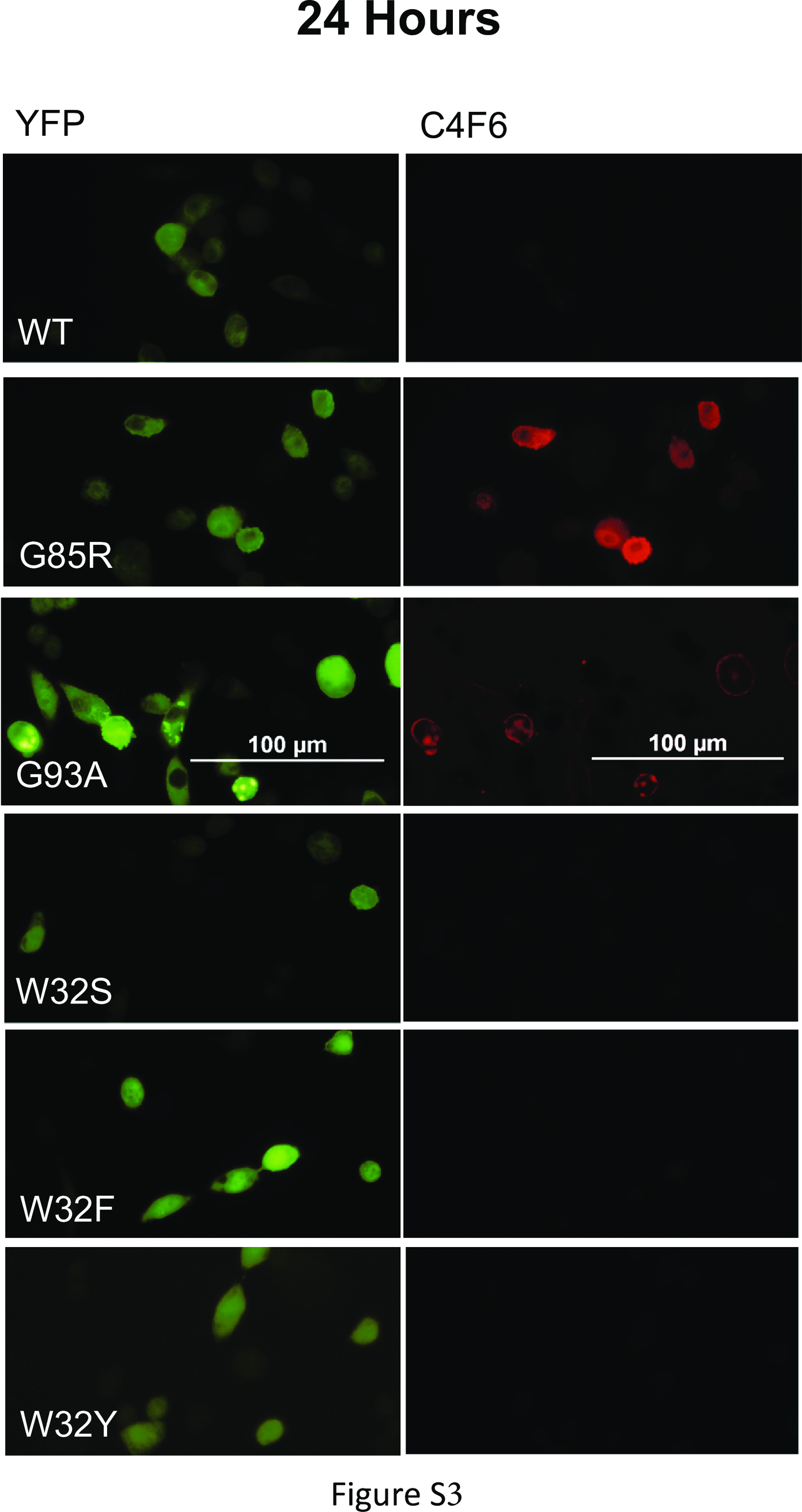

Supplement: S3 Fig — The cells were fixed and stained 24 hours after transfection as described in the methods section. Wild type SOD1: YFP and mutant G85R SOD1: YFP served as a negative and positive controls respectively. The images shown are representative of 3 independent experiments. Mutations on the codon for SOD1 tryptophan position 32 show virtually no C4F6 selectivity, suggesting SOD1 can withstand mutations at tryptophan 32 without misfolding. (TIF) [file pone.0227655.s003.tif]

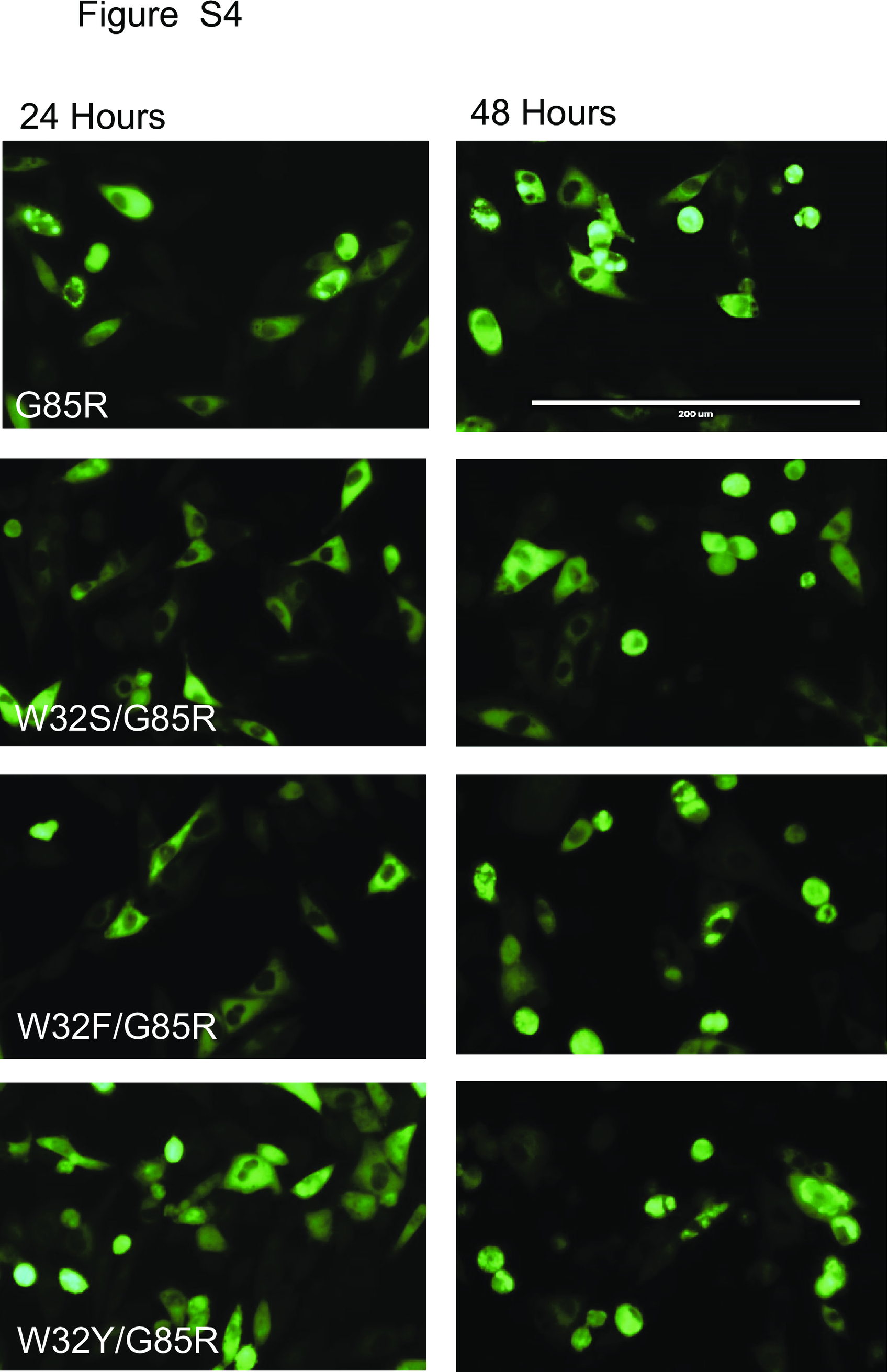

Supplement: S4 Fig — In order to ascertain if mutations at tryptophan 32 are capable of modulating inclusion formation in misfolded SOD1, plasmids encoding the various G85R SOD1: YFP single and double mutations were transiently transfected into CHO cells. Images were taken using fluorescence microscopy at 24 and 48 hours after transfection and subsequently quantified (see Fig 2). The images depicted are representative images from 3 independent experiments. (TIF) [file pone.0227655.s004.tif]

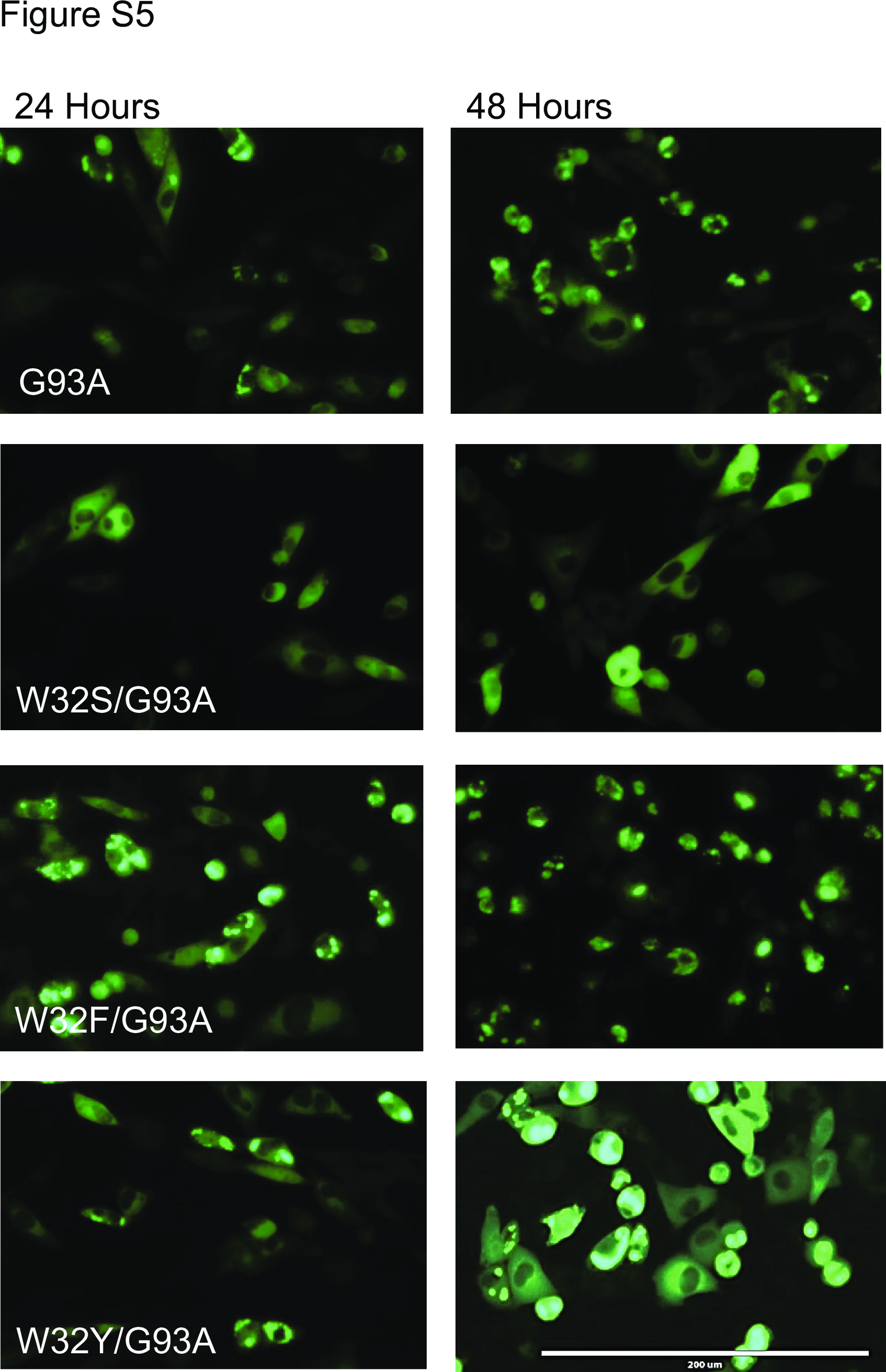

Supplement: S5 Fig — Plasmids for expression of either single or double mutant G93A SOD1: YFP were transiently transfected into CHO cells and then imaged at 24 and 48 hours using fluorescence microscopy. Images were then quantified for changes in tendency for inclusion formation (See Fig 2). The images depicted are representative of 3 independent experiments. (TIF) [file pone.0227655.s005.tif]

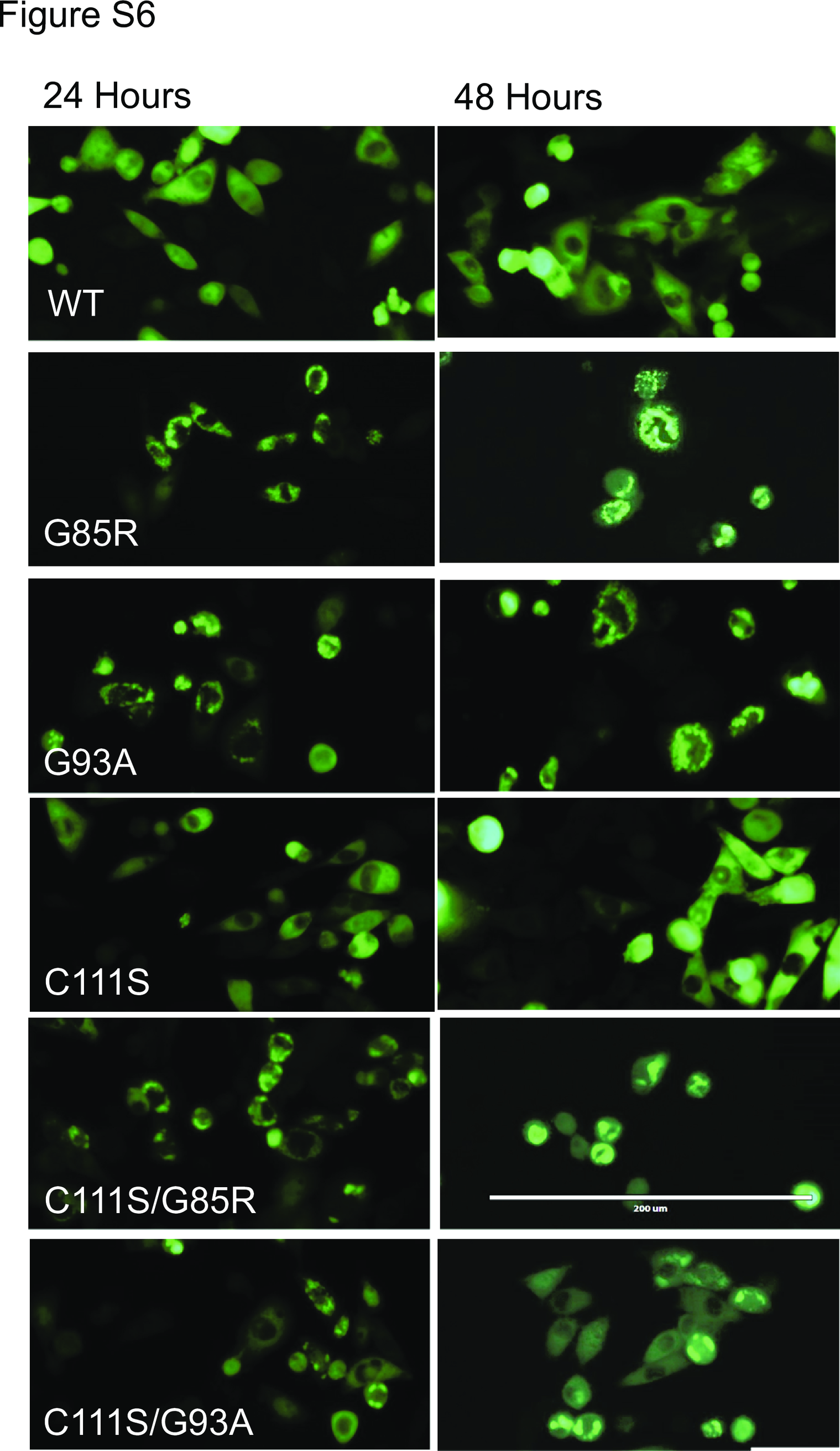

Supplement: S6 Fig — CHO cells were transfected with the various C111S single and double mutant SOD1: YFP cDNA constructs and then imaged 24 and 48 hours after transfection with fluorescence microscopy. Images were then quantified for the presence or absence of aggregates. The images shown are representative images from 3 independent experiments. No observable change in inclusion formation from G85R and G93A single mutant controls was observed. (TIF) [file pone.0227655.s006.tif]

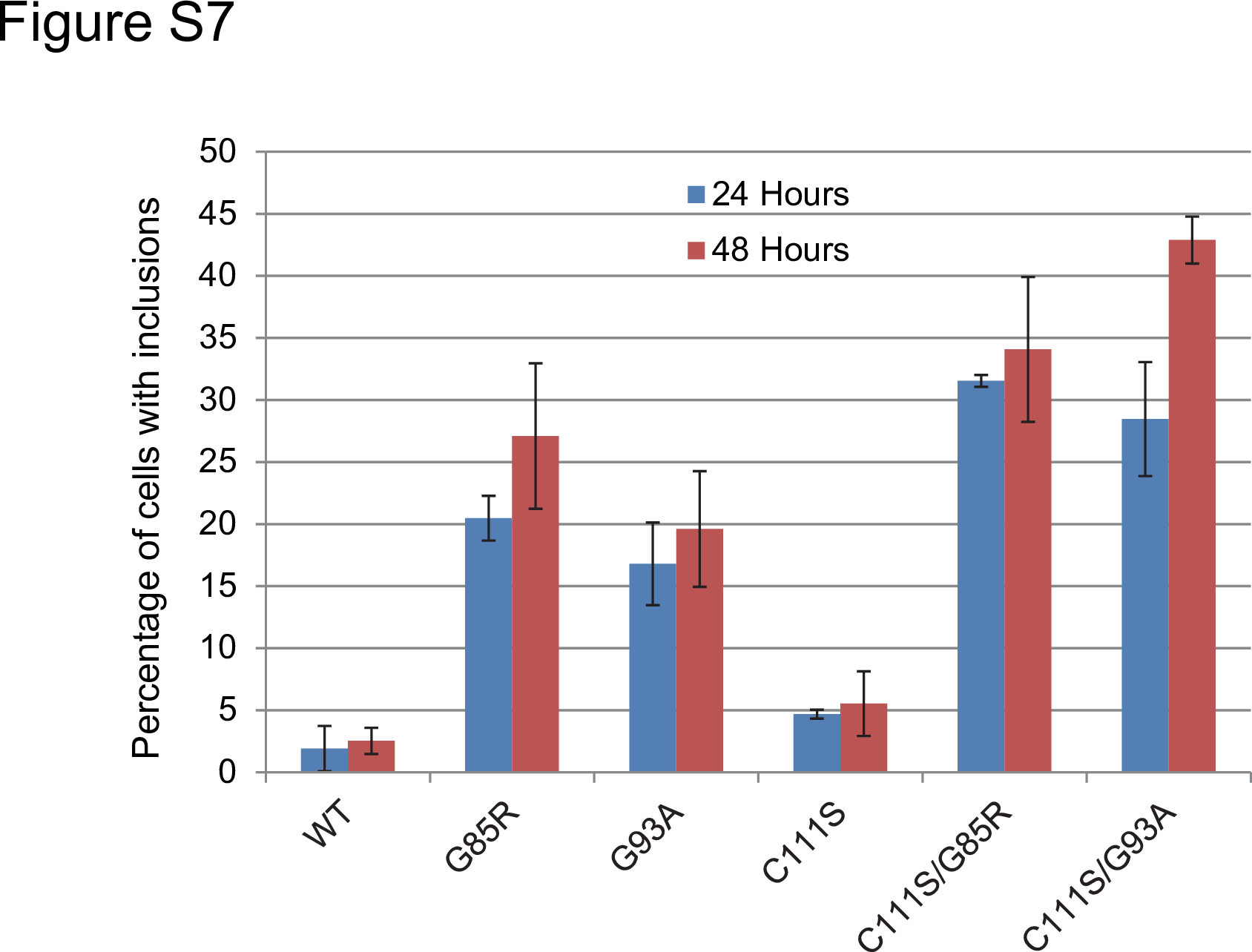

Supplement: S7 Fig — The ability of C111S to suppress aggregation of ALS mutant SOD1 was examined by transient transfection of CHO cells. The data for transfection with WT-, G85R-, and G93A-SOD1: YFP are averages from 6 independent experiments. All other data are from three independent transfections. For all experiments, random images were captured and examined by an observer blind to genotype (S5 Fig for examples of images). The number of total cells counted for each construct across the replications averaged between 79 and 175 cells per construct per experiment. A two-tailed type-2 t-test was used to determine whether the percentage of cells developing inclusions differed between cells expressing individual constructs in a pairwise fashion. The introduction of the C111S substitution to G85R or G93A-SOD1: YFP did not significantly reduce the number of cells that produced inclusions. (TIF) [file pone.0227655.s007.tif]
